# The Daunting Challenge of Ensuring Sustainable Development of Nanomaterials

**DOI:** 10.3390/ijerph13020245

**Published:** 2016-02-22

**Authors:** Mónica J. B. Amorim

**Affiliations:** Department of Biology & CESAM, University of Aveiro, Aveiro 3810-193, Portugal; mjamorim@ua.pt; Tel.: +351-234-247-093

## 1. Introduction

The development and implementation of nanomaterials (NMs) is rapid and a vast range of applications is already in place or foreseen. Consequently, there is a concomitant increased likelihood for NMs being released into the environment. Given this, there is concern regarding the potential ecological sustainability of nanotechnology [1]. Although, this concern has driven an impressive progress in the field of nanotoxicology, there is a considerable need for data that can support fate, exposure and hazard assessment and the modelling thereof.

This Special Issue aimed to tackle the aforementioned requirements by providing a focus on the exposure and effects of NMs in biological systems and the related risk assessment in the environment and for humans. This includes results from novel material and exposure characterisation within various media and biological models; over effect assessment ranging from standard biological endpoints at the population level to subcellular levels, including mechanistic-based studies, to aspects of modelling, risk characterisation, intelligent testing strategies, and safer-by-design approaches.

## 2. Challenges and Prospects

Two decades since the start of NMs Environmental Health and Safety (EHS) studies (e.g., [2]), research has evolved rapidly but challenges persist [3]. The current paradigm of NMs is that the materials characteristics and properties are posing different challenges from those of the traditional chemicals, and also specific requirements in their risk evaluation. In line with this NMs have been given particular attention in terms of regulation and funds [4] in various regions, e.g., in EU [5,6] and in the US [7], by OECD (Organization for Economic Co-operation and Development) WPMN (Working Party on Manufactured Nano-materials). 

Despite the increased focus and progress of NM-related EHS research, several challenges remains for which there are no current solutions, take, for instance, the current lack of proper quantification methods and technology for NMs in test media. Detection and quantification becomes even more difficult when in complex matrixes [8], not to mention that detection limits are often not sufficiently low for detecting materials in environments. The establishment of the required information regarding characterization of NMs has been discussed [9]. This has certain advantages e.g. to account for the possibility of comparability between studies, preferably under standardized procedures (OECD, ISO (International Standard Organization)), and the possibility of future developments and *a posteriori* data completion. 

A fundamental issue in NMs EHS research is the narrowness of available tools, e.g., the most up-to-date existing quantitative technologies, which still only make basic characterization, are available in very few labs worldwide, making it inaccessible for the majority of researchers and referential laboratories. However, focus should be on quantifying the NM relevant and specific biological effects measured (e.g., [10]), regardless of the current impossibility to fully quantify the NMs in media, as long as studies detail the experimental design sufficiently and are carried under standardized procedures or GLP (Good Laboratory Practices). Despite these challenges, inspiring potential lays in high-throughput techniques, such as omics or multi-endpoints systems, where the possibility to understand the mechanisms of action and build up information towards a systems toxicology approach is highlighted [11]. This high-throughput data may also be used for the basis for grouping and read-across approaches [12], approaches which are needed as it is not possible to test each and all NMs in a detailed and timely manner, nor efficient and desired. Coupled with this fast data, longer term exposures to PECs (Predicted Environmental Concentrations) [13] should be carried out, as it may be required to assess effects of NMs and worse case scenarios, longer than the required and established for traditional chemicals. Such new test methods or improvements and adaptations to the existing ones can be very valuable tools in the current framework, e.g., full life cycle tests [14] where additional endpoints and information can be retained and help to discriminate nano effects from non-nano.

Hence, despite the benefits offered by standard methods, efforts should be invested in novel techniques and endpoints, giving the necessary room to explore and the chance to discover the key issues that involve the fate and effect of NMs. As far as to the risk estimations, the most beneficial approach is a flexible and more inclusive approach for NMs Risk Assessment [15]. An intelligent testing strategy (ITS) for NMs is preferred [16].
